# Right Ventricle Thrombus in the Setting of Submassive Pulmonary Embolism

**DOI:** 10.7759/cureus.41161

**Published:** 2023-06-29

**Authors:** Rutul Patel, Anuja Mahesh Mistry, Venkatachalam Mulukutla, Krupal Prajapati

**Affiliations:** 1 Internal Medicine, Texas Tech University Health Sciences Center El Paso, El Paso, USA; 2 Interventional Cardiology, The Hospitals of Providence, El Paso, USA; 3 Internal Medicine, Nathiba Hargovandas Lakhmichand (NHL) Municipal Medical College, Ahmedabad, IND

**Keywords:** submassive pulmonary embolism, right ventricle thrombus, inari, testosterone, anticoagulation, mechanical thrombectomy, right heart strain, thrombus, pulmonary embolism

## Abstract

Submassive pulmonary embolism (PE) with right heart strain and right ventricle thrombus is a complex and potentially life-threatening condition. Prompt recognition and management are crucial to optimizing patient outcomes. We present the case of a 59-year-old male with a history of obstructive sleep apnea (OSA) and non-compliance with continuous positive airway pressure (CPAP) therapy who presented with progressively worsening shortness of breath (SOB). Imaging studies revealed bilateral submassive PE with right heart strain and a thrombus in the right ventricle. Treatment included anticoagulation therapy and mechanical thrombectomy using the INARI FlowTriever device. This case highlights the importance of early recognition and risk factor assessment, such as using testosterone boosters, considering right ventricle thrombus as a complication of PE, and the role of mechanical thrombectomy in selected cases.

## Introduction

Submassive pulmonary embolism (PE) is a severe form of PE associated with significant morbidity and mortality of about 5% to 25% [1]. Risk factors for deep vein thrombosis (DVT) and subsequent PE include immobility, surgery, malignancy, hormonal therapy, and hypercoagulable states. Although less common, right ventricle thrombus affects 4% of all acute pulmonary embolism cases and can further complicate the clinical course and management of PE [2]. We present the case of a 59-year-old male with a history of obstructive sleep apnea (OSA) who presented with progressively worsening shortness of breath (SOB). Despite non-compliance with continuous positive airway pressure (CPAP) therapy, the patient's symptoms warranted further evaluation. Imaging studies revealed bilateral submassive PE with right heart strain and a thrombus in the right ventricle. The patient's recent use of an over-the-counter "testosterone booster" raises the possibility of its association with venous thromboembolism (VTE). The patient underwent a successful mechanical thrombectomy via cardiac catheterization. This case underscores the importance of considering submassive PE in patients with risk factors such as OSA and testosterone use and the potential benefits of mechanical thrombectomy in selected cases, particularly with right ventricle thrombus.

## Case presentation

A 59-year-old male with a past medical history of OSA and non-compliance with CPAP presented to the emergency department (ED) with a complaint of progressively worsening SOB over the past 2 days. The SOB was exacerbated by movement and was particularly severe the night prior to admission. The patient reported no associated chest pain, palpitations, lightheadedness, or fever. There was no history of VTE or prolonged immobilization. The patient mentioned a recent 16-hour vehicle trip approximately two months ago with minimal stops. He denied any recent travel or prolonged immobilization. However, he did admit to using an over-the-counter "testosterone booster" for decreased libido for the past month. Notably, the patient had a known history of OSA but was non-compliant with CPAP therapy.

**Figure 1:**
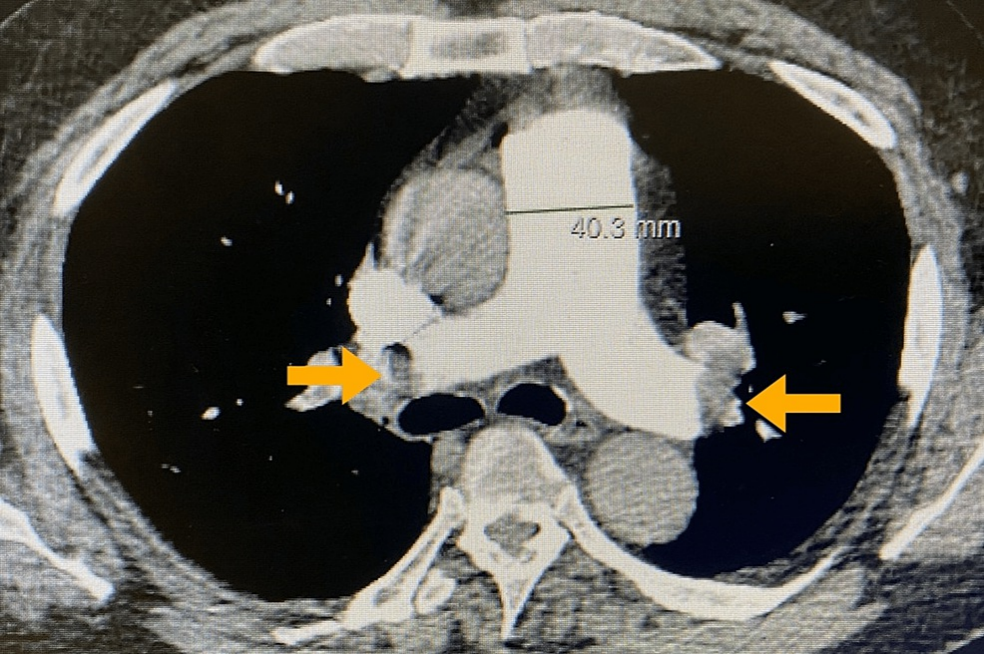
Bilateral pulmonary embolism CTA chest with contrast demonstrates filing defects in bilateral pulmonary arteries indicated by the arrows, suggesting pulmonary embolism. Pulmonary artery diameter is greater than 4 cm, suggesting pulmonary artery aneurysm.

On physical examination, he had tachycardia with a heart rate of 113 beats per minute, a blood pressure of 112/68 mmHg, a respiratory rate of 24/minute, and an oxygen saturation of 93% in room air. Laboratory results showed a significant elevation in D-dimer levels at 9.8 mcg/ml and elevated troponin at 89.9 pg/ml. Contrast-enhanced CT angiography (CTA) of the chest revealed bilateral submassive PE involving the right and left pulmonary arteries, extending into the lobar and segmental branches throughout both lungs (Figure 1). The CTA also indicated right heart strain with an aneurysmal dilatation of the pulmonary artery, straightening of the interventricular septum, and mild clockwise rotation of the cardiac apex. A bilateral lower extremity venous duplex ultrasound revealed a thrombus in the right and left popliteal veins with minimal flow, compatible with incompletely occlusive deep venous thrombosis. His Pulmonary Embolism Severity Index (PESI) score was 90 points, indicating intermediate risk: 3.2-7.1% 30-day mortality. Anticoagulation therapy was initiated with an unfractionated heparin drip infusion and later switched to subcutaneous Enoxaparin (1 mg/kg every 12 hours).

Further assessments were performed, including transthoracic echocardiography (TTE) and transesophageal echocardiography (TEE). TTE revealed a severely dilated right ventricle with an echo-dense mass likely representing a thrombus. The left ventricular chamber size was within normal limits but exhibited mildly reduced systolic function, with an estimated ejection fraction (EF) of 40-45%. The cardiac valves were structurally normal, with mild mitral regurgitation (MR) and tricuspid regurgitation (TR). Pulmonary hypertension was noted, with a pulmonary artery systolic pressure of approximately 55-60 mmHg. The subsequent TEE confirmed the findings of the TTE, including the presence of an intra-atrial septum aneurysm. The TEE revealed the definite presence of an echo-dense mass in the right ventricle, indicating a thrombus (Video 1). The cardiac valves were structurally normal, and the LVEF was estimated to be 40-45%.

**Video 1:** Right ventricle thrombus Transesophageal echocardiogram demonstrating right ventricle thrombus

To further evaluate the right ventricle and address the thrombus, the patient was transferred to the cardiac catheterization laboratory. During the procedure, an echocardiogram performed at the catheterization table revealed the absence of the thrombus, suggesting migration into the pulmonary arteries.

Prior to the intervention, right heart pressures were measured to assess the hemodynamic status of the patient. To gain access to the pulmonary arteries, a Berman wedge catheter was utilized, and a Glidewire was advanced into the right main pulmonary artery. Subsequently, a 22-French sheath was introduced through the right common femoral vein and advanced into the inferior vena cava (IVC). The INARI FlowTriever device was then employed to perform extensive aspiration and mechanical thrombectomy, targeting the right main pulmonary artery and its branches. This intervention resulted in a significant improvement, with a notable reduction in thrombus burden.

After successfully addressing the thrombus in the right pulmonary artery, the catheter was further advanced into the left main pulmonary artery. An angiogram was performed to assess the presence of a thrombus in this area. The procedure was concluded after confirming good flow in the left main pulmonary artery with minimal thrombus.

The patient received post-procedure anticoagulation with Lovenox for two weeks and was subsequently transitioned to full-dose Eliquis. The patient was also educated on the importance of compliance with therapy and lifestyle modifications to reduce the risk of future thrombotic events.

## Discussion

Pulmonary embolism can arise from various etiologies, including DVT and embolization from proximal veins or distant sites. Risk factors for DVT and subsequent PE include immobility, surgery, malignancy, hormonal therapy, and hypercoagulable states [3]. Interestingly, in this case, the patient reported recent use of an over-the-counter "testosterone booster." Testosterone supplementation has been associated with an increased risk of venous thromboembolism, emphasizing the importance of considering potential triggers for PE in clinical evaluation.

Prompt diagnosis of submassive PE is vital, as this condition is associated with significant morbidity and mortality. Imaging modalities such as CTA for the chest play a crucial role in confirming the diagnosis and assessing the extent of the embolism [4]. When patients have a contraindication to intravenous contrast dye, a ventilation/perfusion (V/Q) scan is utilized as an alternative diagnostic modality for PE. In a major study investigating V/Q scanning, namely the Prospective Investigation of Pulmonary Embolism Diagnosis II (PIOPED II) trial, it was found that V/Q scans could reliably exclude PE in patients with a very low probability scan and a very low clinical likelihood of PE [5]. The presence of right heart strain on an EKG or echocardiography can lend support to the diagnosis and provide insights into the severity of the PE. Notably, a distinct echocardiographic finding called McConnell's sign, which is characterized by right ventricular free wall akinesis with sparing of the apex, is highly suggestive of PE [6].

The S1Q3T3 sign, characterized by a prominent S wave in lead I, a Q wave, and an inverted T wave in lead III, serves as an indication of acute cor pulmonale, which signifies right ventricular strain due to pulmonary hypertension [7]. It is important to note that various causes of acute cor pulmonale, including PE, acute bronchospasms, pneumothorax, and other acute lung disorders, can lead to the presence of S1Q3T3 findings on the ECG. During the acute phase of a PE, additional ECG findings may include a new right bundle branch block (complete or incomplete), a rightward shift of the QRS axis, ST-segment elevation in V1 and aVR, generalized low amplitude QRS complexes, atrial premature contractions, sinus tachycardia, atrial fibrillation/flutter, and T-wave inversions in leads V1-V4 [8].

In our case, CTA revealed bilateral submassive PE involving both pulmonary arteries and their lobar and segmental branches. Furthermore, it indicated right heart strain with aneurysmal dilatation of the pulmonary artery, straightening of the intraventricular septum, and mild clockwise rotation of the cardiac apex.

Once diagnosed, the management of submassive PE focuses on preventing further embolization, improving hemodynamics, and reducing the risk of long-term complications. Anticoagulation therapy is the cornerstone of treatment and is initiated promptly to prevent clot propagation. In this case, the patient received an unfractionated heparin drip infusion, followed by subcutaneous enoxaparin. Currently, preferred options for anticoagulation include oral factor Xa inhibition with agents such as apixaban, rivaroxaban, and dabigatran, which do not require frequent laboratory monitoring [9].

In addition to anticoagulation, various treatment options exist for submassive PE, depending on the patient's clinical presentation and hemodynamic stability. Thrombolytic therapy can be considered in selected cases to rapidly dissolve the thrombus and improve hemodynamics. The mortality benefits of systemic thrombolysis in intermediate-risk PE have been a subject of debate in various studies and meta-analyses, with conflicting results. Although some studies have shown short-term benefits, there is a significant association with increased bleeding, including intracranial hemorrhage, particularly in older patients [10].

To mitigate the risk of major bleeding, alternative approaches such as reduced-dose thrombolysis and catheter-based interventions have been explored. Mechanical thrombectomy has emerged as an alternative treatment modality for submassive PE [11]. It offers the advantage of directly removing the thrombus, thereby rapidly improving hemodynamics. The INARI FlowTriever device, used in our case, facilitates extensive aspiration and mechanical thrombectomy of the thrombus burden. This percutaneous approach has shown promising results and provides a viable option for patients who are not candidates for or have contraindications to thrombolytic therapy.

Saddle embolism, defined as an embolus lodged at the bifurcation of the main pulmonary artery, is a high-risk variant of PE that requires immediate intervention if associated with pre-existing cardiopulmonary disease [12]. It can cause severe hemodynamic compromise and even cardiac arrest. Prompt diagnosis and management are crucial to preventing fatal outcomes. In our case, the imaging studies did not reveal a saddle embolism, but the patient presented with bilateral submassive PE involving the right and left pulmonary arteries.

A thrombus within the right ventricle poses an additional concern, as it can lead to further embolization or right ventricular dysfunction. Treatment options for right ventricle thrombus include anticoagulation therapy and consideration of interventions such as mechanical thrombectomy or surgical embolectomy [13]. In our case, the presence of an echo-dense mass in the right ventricle on TTE and confirmation on TEE prompted the decision to proceed with mechanical thrombectomy using the INARI FlowTriever device. This intervention resulted in a significant reduction in the thrombus burden and an improvement in the patient's condition.

## Conclusions

In conclusion, bilateral submassive pulmonary embolism with right heart strain and right ventricle thrombus requires a comprehensive approach to management. Identifying potential triggers for pulmonary embolisms, such as the use of testosterone boosters, is crucial in the evaluation of patients. Prompt diagnosis through imaging studies and appropriate risk stratification guide the selection of treatment options. Anticoagulation remains the cornerstone of therapy, with thrombolytic therapy and mechanical thrombectomy serving as potential interventions in selected cases. This case highlights the importance of a multidisciplinary approach, involving cardiology, pulmonology, and interventional radiology, to optimize patient outcomes and prevent long-term complications associated with submassive pulmonary embolism and right ventricle thrombus formation.
